# A laboratory evaluation of alcohols as attractants for the sandfly *Lutzomyia longipalpis* (Diptera:Psychodidae)

**DOI:** 10.1186/1756-3305-7-60

**Published:** 2014-02-06

**Authors:** Jairo Torres Magalhães-Junior, Stella Maria Barrouin-Melo, Arlene Gonçalves Corrêa, Flavia Benini da Rocha Silva, Vicente Estevam Machado, José Silvio Govone, Mara Cristina Pinto

**Affiliations:** 1 Departamento de Anatomia, Patologia e Clínicas Veterinárias, Escola de Medicina Veterinária e Zootecnia, Universidade Federal da Bahia, Av. Ademar de Barros 500, 40170-000 Salvador, BA, Brazil; 2 Departamento de Química, Universidade Federal de São Carlos, 13565-905 São Carlos, SP, Brazil; 3 Departamento de Ciências Biológicas, Faculdade de Ciências Farmacêuticas, Universidade Estadual Julio de Mesquita Filho, UNESP, 14801-902 Araraquara, SP, Brazil; 4 Departamento de Estatística Matemática Aplicada E Computação Universidade Estadual Paulista Júlio de Mesquita Filho, Instituto de Geociências e Ciências Exatas de Rio Claro, v 24 A, 1515, Bela Vista, 13506-970 Rio Claro, SP, Brazil

**Keywords:** Sandflies, Phlebotomine, Kairomone, Octenol, Octanol, Heptanol, Nonanol, Attraction, Wind tunnel

## Abstract

**Background:**

The potential attraction from 1-octen-3-ol for sandflies has been documented; however, studies using other primary alcohols are limited.

**Findings:**

We used a wind tunnel to compare the activation and attractive behaviors in male and female *Lutzomyia longipalpis* using 1-octen-3-ol and three additional alcohols, 1-octanol, 1-heptanol and 1-nonanol at three different concentrations: neat (100%) and diluted in hexane (10% and 50%). The compounds 1-octen-3-ol and 1-nonanol induced a clear concentration-dependent activation and attraction response in females. In males, 1-octen-3-ol, 1-nonanol and 1-heptanol yielded the same results.

**Conclusions:**

*L. longipalpis* is attracted to 1-octen-3-ol, 1-nonanol and 1-heptanol, which are found in many plant volatiles.

## Findings

### Background

Volatile compounds used as haematophagous insect lures may improve the efficacy of traps for surveillance and control of disease vectors. For the sandfly *Lutzomyia longipalpis* (Lutz & Neiva), which is the major *Leishmania infantum* vector in South America, previous investigations into attractive lures have focused on male pheromones [1] and kairomones [2].

The kairomone 1-octen-3-ol (hereafter octenol) is a volatile component of bovine [3] and human breath [4]. Its potential role as an attractant has been documented for different haematophagous insect species, such as mosquitoes [5] and tsetse flies [3]. For the New World sandfly species, octenol has previously been used with light traps and found to be relatively attractive to *Psathyromyia shannoni* (Dyar) (=*Lutzomyia shannoni*) [6] and, in a concentration-dependent manner, to *Nyssomyia neivai* (Lutz & Neiva) (=*Lutzomyia intermedia*) [7]. For *L. longipalpis*, octenol elicited significant olfactory responses in electrophysiological experiments [8], but it showed a weak attractive response at 0.5 mg/h associated with light traps under field conditions [2].

Unlike octenol, studies on the potential attractiveness of other primary alcohols, namely, 1-octanol, 1-heptanol, 1-nonanol (hereafter, octanol, heptanol and nonanol), for haematophagous insects are limited. These alcohols were identified at small levels in incubated human sweat [9]. Only nonanol has been demonstrated as relatively attractive to *Aedes aegypti* (Linnaeus) compared with a control [10]. No studies have been reported on attractiveness of these alcohols to sandflies.

The aim of this study was to evaluate *L. longipalpis* male and female responses to octenol, octanol, heptanol and nonanol at different concentrations using the wind tunnel method.

### Methods

#### Insects

Sandflies were collected in Ipecaetá (12º18’00’S 39º18’28”W), Bahia State and kept in a colony at the Laboratory of Veterinary Infectious Diseases (Federal University of Bahia) for 18 generations. The insects were maintained in netting cages using standard methods with access to a 50% sucrose solution at 26 ± 1°C and 80–90% humidity. The sandfly species names are presented using the Galati classification system [11] followed by the corresponding Young and Duncan nomenclature [12] in brackets when cited for the first time.

#### Bioassay protocol in the wind tunnel

Each bioassay was performed from 9:00 to 19:00 in a transparent acrylic wind tunnel (length 200 cm, width 20 cm and height 20 cm) as previously described [13]. For each test, three male or female *L. longipalpis* were placed inside a releasing chamber for 30 min for acclimation before each test. The insects were 3-6 days old and received only sugar meal. Females had not received a blood meal. The chamber was then placed inside the wind tunnel 50 cm downwind from the odor source. Each trial was 2 min long, and we recorded the sandfly activation and attraction behaviors. The activation behavior was demonstrated through the number of sandflies that left the releasing chamber. The attraction behavior was demonstrated through the number of sandflies that reached the odor source. Thirty insect specimens were used for each concentration per compound.

The compounds used for the experiments were octenol, octanol, heptanol and nonanol (98.0%, Aldrich Chemical, Milwaukee, WI) at three different concentrations: neat (100%) and diluted in hexane (10% and 50%). Each concentration was released by placing 200 μL onto filter paper (4 × 4 cm) in the wind tunnel entrance. The controls were 200 μL of hexane on filter paper (4 × 4 cm) before each trial.

#### Statistical analysis

Chi square tests were used to evaluate the different proportions of males and females activated and attracted by each compound. Initially, the test was conducted for all four groups simultaneously. Thereafter, if a significant difference was verified, each of the two groups was compared separately. The statistical analyses were performed using BioEstat (version 5.0; Mamirauá/CNPq, Belém, PA, Brazil).

### Results

#### Female responses

**Figure 1:**
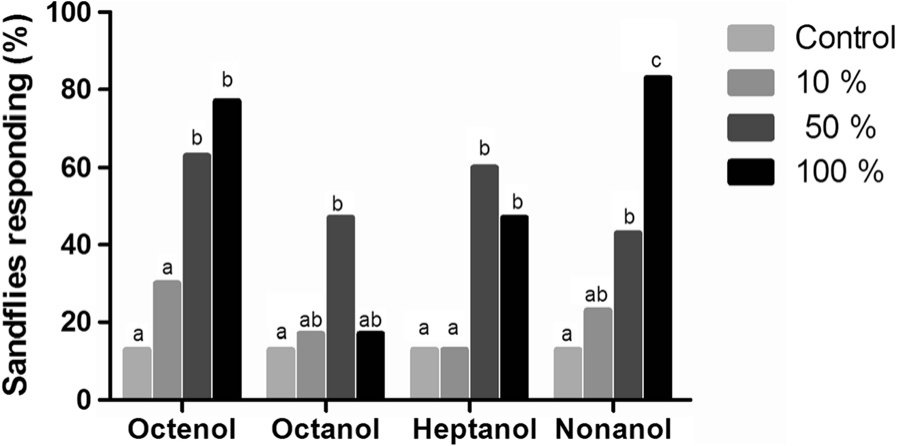
**Activation of female *L. longipalpis*.** Percentage of female *L. longipalpis* activated by octenol, octanol, heptanol and nonanol (three different concentrations) in the wind tunnel. Bars with different letters were significantly different in pairwise comparisons (p < 0.05).

**Figure 2:**
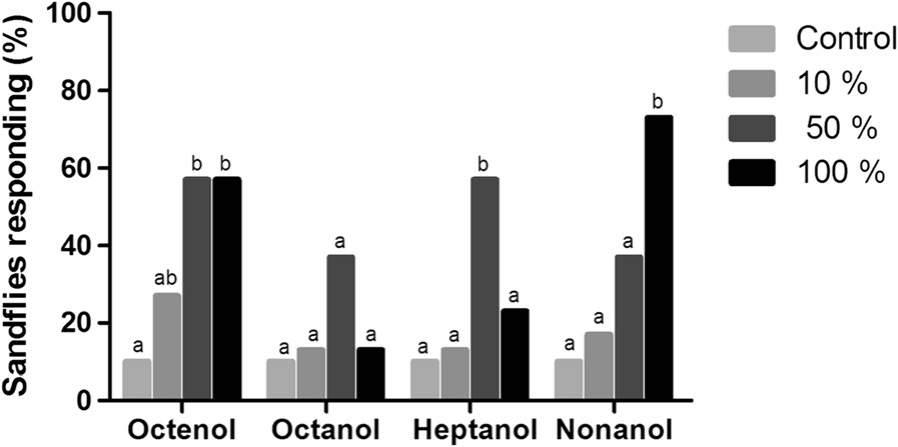
**Attraction of female *L. longipalpis*.** Percentage of female *L. longipalpis* attracted by octenol, octanol, heptanol and nonanol (three different concentrations) in the wind tunnel. Bars with different letters were significantly different in pairwise comparisons (p < 0.05).

Octenol and nonanol induced a clear concentration-dependent activation and attraction response within the dosage range evaluated. For octenol, the activation and attraction responses were significantly different at the 50% concentration compared with the control (p < 0.05), but the 50% and 100% concentrations were not different (p > 0.05). For nonanol, the activation response was statistically different from the 50% concentration compared with the control; however, there was a significantly different attraction response only at the 100% concentration (p < 0.05) (Figures 1 and 2).

The female activation and attraction response to octanol did not yield a concentration-dependent pattern. The only statistical difference detected was for activation at the 50% concentration compared with the control (p < 0.05).

For heptanol, the female activation responses were concentration-dependent; the 100% response was greater than at 50% compared with the control (p < 0.05). However, only the 50% concentration yielded a significantly different insect attraction response compared with the control (p < 0.05); the 100% concentration was not different from the control (p > 0.05).

#### Male responses

The male sandfly responses followed a similar pattern as the females for octenol, nonanol and heptanol (i.e., a concentration-dependent response).

**Figure 3:**
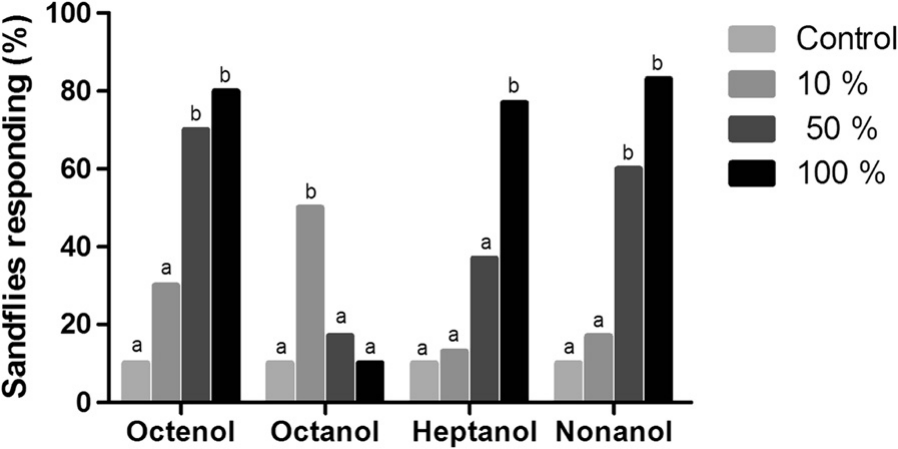
**Activation of male *L. longipalpis*.** Percentage of male *L. longipalpis* activated by octenol, octanol, heptanol and nonanol (three different concentrations) in the wind tunnel. Bars with different letters were significantly different in pairwise comparisons (p < 0.05).

**Figure 4:**
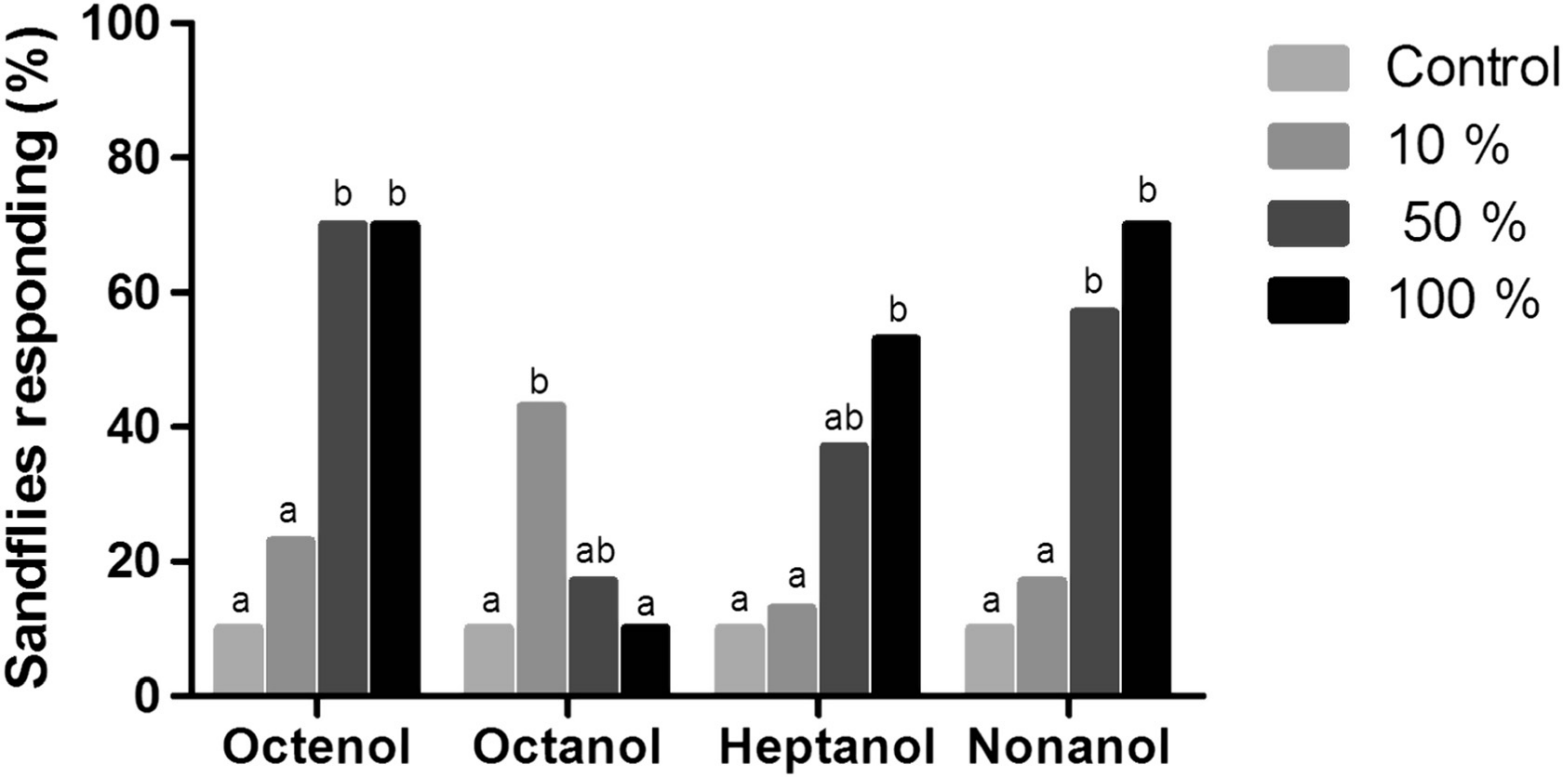
**Attraction of male *L. longipalpis*.** Percentage of male *L. longipalpis* attracted by octenol, octanol, heptanol and nonanol (three different concentrations) in the wind tunnel. Bars with different letters were significantly different in pairwise comparisons (p < 0.05).

For octanol, similar to the females, the males did not exhibit a concentration-dependent response; however, the only significant difference detected in the activation and attraction response was at the 10% concentration compared with the control (p < 0.05). For heptanol, although the males presented the same pattern as the females, the best activation and attraction responses were at the highest concentration (100%) compared with the control (p < 0.05) (Figures 3 and 4).

### Discussion

Our results show that in addition to octenol, other alcohols evoke sandfly responses and should also be investigated. Octenol and nonanol elicited the highest responses from *L. longipalpis* females, and octenol, heptanol and nonanol elicited the highest responses from males. Further, not all mosquito species respond equally to octenol [5], and whether different groups of alcohols would increase attractant activity for such species has been discussed [10]. However, a clear structure-activity relationship has not been demonstrated [14].

Octenol’s role as a kairomone has been extensively evaluated in haematophagous insects because it is found in different sources, such as bovine [3] and human breath [15]. Octenol’s role in attracting sandflies is controversial, but our results showed a clear dose-dependent response with weak attractiveness at a low concentration (10%). These results may explain the poor results from *L. longipalpis* captures in the field when octenol was used at a low concentration (0.5 mg/h) [2].

Nevertheless, primary alcohols, such as octanol, heptanol and nonanol, have not been well-investigated for haematophagous insects, and this is the first report of such a study using sandflies. Such alcohols are not directly associated with vertebrate breath or skin odors, which may be the basis for the lack of interest in their potential role as an attractant for disease vectors. Nonetheless, those alcohols were detected at small levels in human sweat after incubation for 42-52 h, and nonanol presented the highest levels compared with octanol and heptanol [9]. Heptanol was also observed in chicken feather hydrolysate [16], which is relevant for sandflies because they are present at high levels in chicken sheds. Such bird shelters are the putative breeding sites for *L. longipalpis*[17]. The literature reports on haematophagous insect responses to octanol, heptanol and nonanol are summarized in Table 1[18-20].

**Table 1:** Reports of haematophagous insect responses to octanol, heptanol and nonanol

**Compounds**	**Sources of compounds**	**Insects**	**References**
		**Experimental design responses**	
1-octanol	Commercial (Aldrich)	*Simulium arcticum*	Sutcliffe *et al*. [18]
		Field Negative attractiveness	
1-heptanol	Metasternal glands of *Triatoma brasiliensis*	*Triatoma brasiliensis*	Vitta *et al*. [19]
		CG- EAD	
		No response	
1-nonanol	Volatiles from cattle headspace and urine	*Haematobia irritans Stomoxys calcitrans*	Birkett *et al*. [20]
		CG-EAG Positive response	

Floral volatiles are composed of various substances that have been shown to be attractive to mosquitoes [21]. The primary alcohols herein were identified in several mushrooms species [22] and other herbaceous plants [23]. From an environmental perspective, it is noteworthy that primary alcohols are in plants, which are generally sandfly feed sources. Both male and female sandflies require carbohydrates for energy, which are acquired through feeding directly on plant tissues in the field [24].

Herein, we observed similarities and distinctions between the insect sexes considering their biological responses to the primary alcohols evaluated. Although both males and females require plant sap to survive and specific variation in attraction to their constituent compounds is expected, it is difficult to explain such events through reports on morphological aspects. Differences were also observed in the number of sensilla on the second palpal segment in females (2-6) compared with males (1-2). However, an equal number of sensilla were observed in the third segment of the maxillary palps (Newstead’s sensilla) for *L. longipalpis* males and females [25].

A dose-dependent response to octenol in female *L. longipalpis* was previously observed through electrophysiological recordings [8]. The female behavioral responses to octenol herein are consistent with such studies. Although no studies have investigated male electrophysiological responses to octenol, they were both activated and attracted in the wind tunnel.

Plant-specific emissions are important for attracting sandflies, and further studies with plant volatiles may be a potential approach to improve sandfly lures.

## Competing interests

The authors declare that they have no competing interests.

## Authors’ contributions

JMJ, SM, AC and MP conceived the study and drafted the manuscript. JMJ, MP, FS and VM conducted the bioassay experiments. JG provided statistical analysis. All authors read and approved the final manuscript.
